# Pooling Upper Respiratory Specimens for Rapid Mass Screening of COVID-19 by Real-Time RT-PCR

**DOI:** 10.3201/eid2610.201955

**Published:** 2020-10

**Authors:** So Yeon Kim, Jaehyeon Lee, Heungsup Sung, Hyukmin Lee, Myung Guk Han, Cheon Kwon Yoo, Sang Won Lee, Ki Ho Hong

**Affiliations:** National Medical Center, Seoul, South Korea (S.Y. Kim);; Jeonbuk National University Medical School and Hospital, Jeonju, South Korea (J. Lee);; Asan Medical Center and University of Ulsan College of Medicine, Seoul (H. Sung);; Yonsei University College of Medicine, Seoul (H. Lee);; Korea Centers for Disease Control and Prevention, Cheongju (M.G. Han, S.W. Lee, C.K. Yoo);; Seoul Medical Center, Seoul (K.H. Hong)

**Keywords:** coronavirus disease, SARS-CoV-2, severe acute respiratory syndrome coronavirus 2, severe acute respiratory syndrome, SARS, viruses, respiratory infections, zoonoses, COVID-19, SARS-related coronavirus, COVID-19, SAR-CoV-2, real-time PCR, reverse transcription PCR, specimen pooling, South Korea

## Abstract

To validate the specimen-pooling strategy for real-time reverse transcription PCR detection of severe acute respiratory syndrome coronavirus 2, we generated different pools including positive specimens, reflecting the distribution of cycle threshold values at initial diagnosis. Cumulative sensitivities of tested pool sizes suggest pooling of <6 specimens for surveillance by this method.

After the first report of the coronavirus disease (COVID-19) outbreak in Wuhan, China (*1*), the World Health Organization announced pandemic status on March 11, 2020 (*2*). Real-time reverse transcription PCR (rRT-PCR) detection of the causative agent, severe acute respiratory syndrome coronavirus 2 (SARS-CoV-2), is a confirmatory diagnostic tool for COVID-19 (*3*).

A mass screening test for COVID-19 is urgently needed in South Korea because of the increasing number of confirmed cases in long-term care hospitals and public facilities, as well as imported cases. Testing specimens pooled before RNA extraction and subsequently retesting single specimens from positive pools is an efficient strategy for rapid mass screening as well as for increasing testing capacity and conserving resources.

Testing pooled specimens is a well-known method and has been used in blood banks worldwide to screen for infectious disease; however, only a few studies have evaluated specimen pooling for SARS-CoV-2 (*4*,*5*; R. Hanel et al., unpub. data. https://arxiv.org/abs/2003.09944v1; M.J. Farfan et al., unpub. data, https://doi.org/10.1101/2020.04.15.20067199). Therefore, we evaluated the pooling strategy for SARS-CoV-2 testing using clinical specimens from 3 hospitals in South Korea: Seoul Medical Center and National Medical Center, both in Seoul, and Jeonbuk National University Hospital in Jeonju. The Institutional Review Boards of the hospitals approved this study. Written consent from participants was waived.

## The Study

Pooled upper respiratory specimens were prepared from 50 individual SARS-CoV-2–positive specimens and 300 individual SARS-CoV-2–negative specimens. Either a single nasopharyngeal swab (NPS) or a nasopharyngeal and an oropharyngeal swab (NPS/OPS) were collected in an eNAT tube (Copan Italy, https://www.copangroup.com). Laboratory diagnosis of SARS-CoV-2 infection was performed with all specimens using the following rRT-PCR kits targeting the *E* and *RdRp* genes: STANDARD M nCoV Real-time Detection (SD Biosensor, https://sdbiosensor.com) or PowerCheck 2019-nCoV Real-Time Detection (Kogene Biotech, https://www.kogene.co.kr).

**Figure 1:**
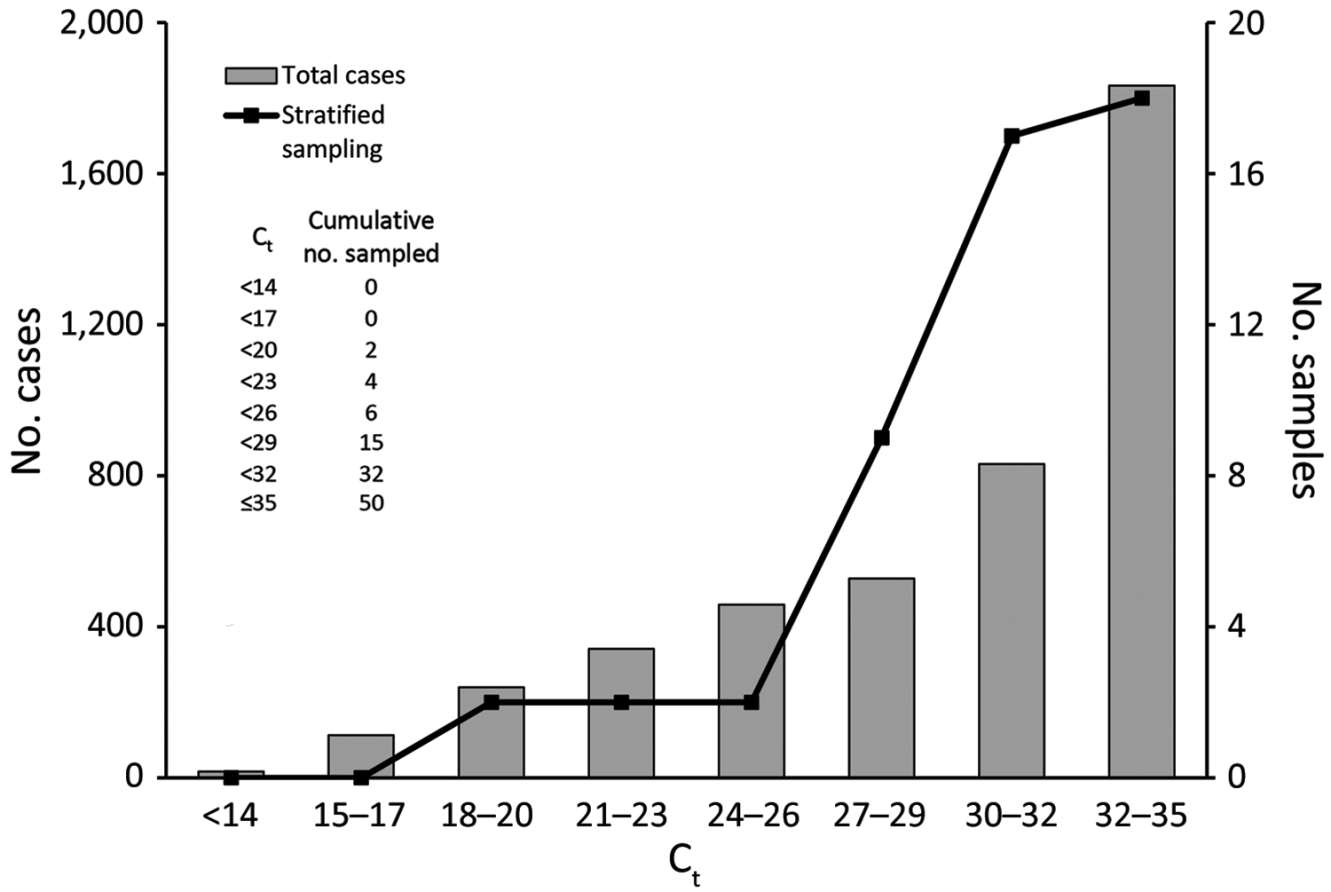
Distribution of *RdRp* gene (C_t_) values for specimens from 4,364 confirmed patients in South Korea at their initial diagnosis of coronavirus disease (COVID-19) and the specimens selected by stratified sampling. This figure shows the first *RdRp* gene C_t_ values of patients receiving a COVID-19 diagnosis (bars). We selected positive samples with the stratified sampling method based on that distribution (line). Cumulative numbers of selected specimens per stratum are shown. C_t_, cycle threshold.

For the SARS-CoV-2–positive pooled specimens, we selected 50 individual SARS-CoV-2–positive specimens on the basis of the observed population distribution of cycle threshold (C_t_) values of r RT-PCR for patients confirmed positive during January 20–March 2, 2020 (Figure 1). We grouped the C_t_ values into 8 strata, decided the sampling number adequate for each stratum, and selected a total of 50 specimens for 8 strata (Figure 1). We pooled the selected individual SARS-CoV-2–positive specimens with different numbers of SARS-CoV-2–negative specimens to generate 50 sets of pooled specimens in duplicate; the pool sizes of each set were 2, 4, 6, 8, 10, and 16. We prepared a total of 600 pooled specimens. To evaluate clinical specificity in SARS-CoV-2–negative pooled specimens, we randomly combined 16 specimens from 300 negative specimens and generated 60 negative pooled specimens (Appendix).

The following 3 automated RNA extraction systems were used: MagNa Pure 96 (Roche Diagnostics, https://www.roche.com), Real-prep (BioSewoom, www.biosewoom.com), and eMAG (bioMérieux, https://www.biomerieux.com). We followed the extraction protocol provided by each manufacturer with an input volume of 200 μL and elution volume of 50 μL.

We performed rRT-PCR using PowerCheck 2019-nCoV for all pooled specimens. The interpretation guideline by the manufacturer for SARS-CoV-2 positivity was a C_t_ cutoff of <35 for a single specimen; however, we assessed every amplified curve throughout 40 total PCR cycles. For either the *E* or *RdRp* gene, when we observed any amplified curve before the end of the 40 amplification cycles, we interpreted the result as positive for the pooled specimens. When we observed no amplification curves for both genes, we interpreted the result as negative.

We performed all statistical analyses with MedCalc version 19.2.1 (MedCalc Software Ltd, https://www.medcalc.org). The distribution of C_t_ values in individual specimens (Figure 1) showed negative skewness. In total, 61% of confirmed cases had C_t_ >30, which was near the cutoff value. We selected positive samples for pooling according to this distribution pattern.

**Figure 2:**
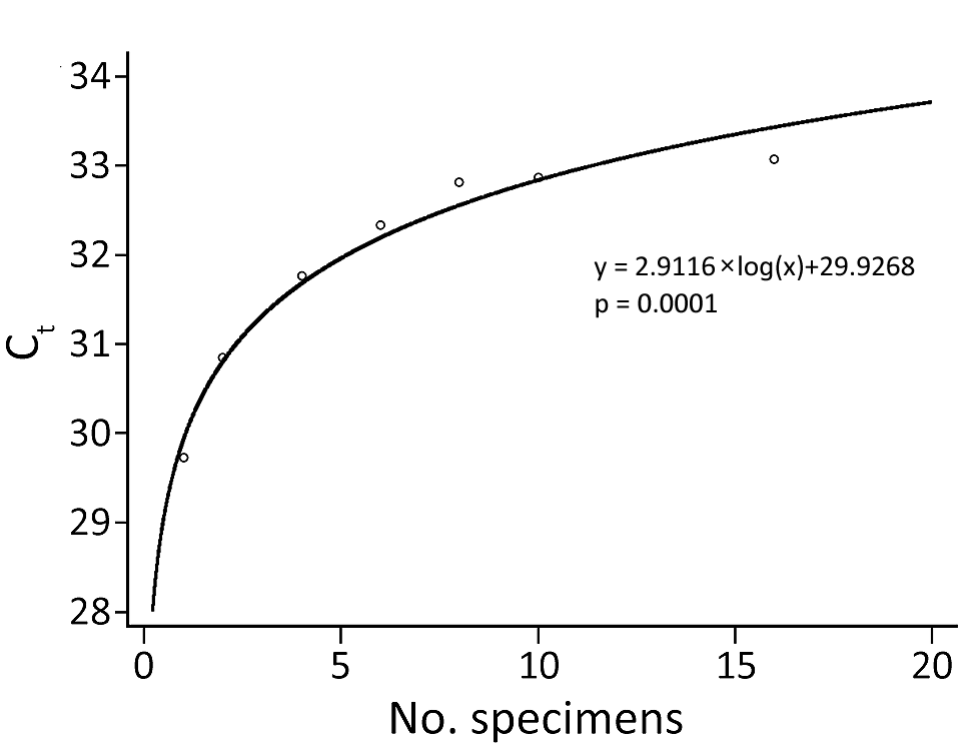
Mean C_t_ values of *RdRp* genes of 50 specimens from coronavirus disease patients in South Korea by pool size. The trend line shows logarithmic regression. C_t_, cycle threshold.

The pooled positive specimens had 100% sensitivity in pool sizes 2, 4, and 6 and 97%–99% sensitivity in pool sizes 8, 10, and 16 (Table). To ensure a conservative estimation of sensitivity, we calculated the cumulative sensitivities on the assumption that the false-negative results that occurred in smaller pool sizes could also occur in larger pool sizes. Therefore, every negative result that occurred in smaller pool sizes was included in the calculation of cumulative sensitivities in larger pool sizes. The cumulative sensitivities of pool size 6 was 100%, of 8, 97%, of 10, 96%, and of 16, 92%. The clinical specificity of pool size 16 was 97% (58/60, 95% CI 87%−99%). The mean C_t_ values increased for both the *E* and *RdRp* genes as the pool size increased (Figure 2; Appendix Figure). 

**Table:** Test performance of pooled specimens compared with individual specimens for severe acute respiratory syndrome, virus 2

No. specimens in pool	Amplification in *E* or *RdRp* gene, %	No amplifications	Sensitivity of pools, % (95% CI)	Cumulative sensitivity, %*
2	100	0	100 (96–100)	100
4	100	0	100 (96–100)	100
6	100	0	100 (96–100)	100
8	97	3	97 (92–99)	97
10	99	1	99 (95–100)	96
16	96	4	96 (90–98)	92

*Calculated sensitivity based on the accumulated discrepancy numbers under the dilution fold

## Conclusions

We evaluated the clinical sensitivity and specificity of SARS-CoV-2 rRT-PCR using pooled upper respiratory specimens from confirmed cases. Because pooled specimens are expected to be used as a screening tool, the clinical sensitivity of pooled specimens at a given pool size is especially important.

A limitation of previous studies is that the C_t_ values of positive specimens from patients at the time of diagnosis were not considered in the study design. The C_t_ values of specimens in previous studies were relatively low (*6*). Because specimens with high C_t_ values, meaning low virus titers, are expected to be vulnerable to pooling, the distribution of C_t_ values in the actual population should be reflected when determining the pool size. We analyzed the actual distribution of C_t_ values from 4,364 initially confirmed cases, and the distribution showed skewness with regard to the PCR cutoff value.

Yelin et al. (*4*) suggested that the pool size using RNA extracts could be <64; however, we do not recommend increasing the pool size to 64, corresponding to a theoretical increase in C_t_ values of 6, given the associated loss in sensitivity; doing so may cause false negative results.

The pooling strategy showed efficiency when the positive rates in the population were low (*7*). We showed clinical sensitivities and cumulative sensitivities of the pooled specimens that were sampled after stratification by data, including low viral titers. On the basis of our results, we recommend pooling <6 specimens in clinical practice. Pooling >6 specimens might cause false-negative results, considering the observed abundance of specimens with high C_t_ values in the population.

This study has some limitations. First, the analytical performance of the PCR kit used has not been evaluated fully because it is one of the earliest available commercial PCR kits that received the Emergency Use Authorization in Korea. Second, the positive cutoff in the kit was a C_t_ value <35 within 40 amplification cycles. Therefore, this study did not include individual specimens with a C_t_ value >35, which is interpreted as an inconclusive result by this kit. Third, we did not evaluate cost-effectiveness on the basis of the hypothesized prevalence. Last, we did not evaluate the effect of specimen volume in the pools; increasing the input volume from each specimen may improve the sensitivity of the pooling test.

Our protocol will be helpful for screening persons in groups at high risk for COVID-19 infection quickly and quarantining those confirmed positive, even in situations with limited time and test resources. Epidemiologic factors should be considered when choosing an adequate pooling number. Symptomatic case-patients should be tested individually without pooling to enable effective and timely action. We have included practical guidelines for specimen-pooling procedures in the Appendix.

Appendix Additional information about sampling from pooled specimens for rapid mass screening for coronavirus disease.
