# Supplementary material for: Pooling Upper Respiratory Specimens for Rapid Mass Screening of COVID-19 by Real-Time RT-PCR
Source: Emerg Infect Dis. 2020 Oct;26(10):2469–72. doi: 10.3201/eid2610.201955 (PMC7510748; doi:10.3201/eid2610.201955)
Supplement: Appendix — Additional information about sampling from pooled specimens for rapid mass screening for coronavirus disease. [file 20-1955-Techapp-s1.pdf]

Article DOI: https://doi.org/10.3201/eid2610.201955

Pooling Upper Respiratory Specimens for Rapid Mass Screening of COVID-19 by Real-Time RT-PCR

Appendix

Authors' Protocol for Molecular Testing for COVID-19 with Pooled Upper Respiratory Specimens

1. Background

Real-time reverse-transcription PCR (RT-PCR) testing is the standard testing modality for coronavirus disease 2019 (COVID-19). As of April 7, 2020, 470,000 persons have been tested, of which, more than 10,000 individuals were confirmed to be positive. In principle, attempts are made to collect specimens from both the upper and lower respiratory tract specimens are tested individually: a combination of oropharyngeal/nasopharyngeal swab and sputum (*1*,*2*).

The estimated testing capacity in South Korea is approximately 30,000 specimens per day. However, with an increase in the number of new cases from group facilities, foreign entrants, and long-term care facilities, the need for large-scale testing that targets an entire group or population has been suggested. The test with pooled samples, a method widely used to screen viruses in donated blood, is one of the mass screening strategy. This has been attempted in some countries for COVID-19 and in a selected population in South Korea.

The COVID-19 Task Force of the Korean Society for Laboratory Medicine (KSLM) and the Center for Laboratory Control of Infectious Diseases of Korean Centers for Disease Control and Prevention (KCDC) have proposed a protocol for molecular testing for COVID-19 with pooled upper respiratory specimens, based on currently available data and on the results of validation studies using clinical specimens. This protocol is intended for use at public laboratories and at laboratories in medical institutions. This protocol may be modified based on additional studies or data.

2. Materials and Methods for Protocol Development

a. Specimen selection and preparation of pooled specimens

Pooled upper respiratory specimens were prepared from 50 individual SARS-CoV-2-positive specimens and 300 individual SARS-CoV-2-negative specimens. Either a single nasopharyngeal swab (NPS) or 2 nasopharyngeal/oropharyngeal swabs (NPS/OPS) were collected in an eNAT tube (Copan Italy, https://www.copangroup.com). Laboratory diagnosis of SARS-CoV-2 infection was performed with all specimens using the following real-time RT-PCR kits targeting the *E* and *RdRp* genes: STANDARD M nCoV Real-time Detection (SD Biosensor, sdbiosensor.com) or PowerCheck 2019-nCoV Real-Time Detection (Kogene Biotech, https://kogenebiotech.abraa.com).

For the SARS-CoV-2-positive pooled specimens, 50 individual SARS-CoV-2-positive specimens were selected based on the distribution of observed threshold cycle (C_t) values of real-time RT-PCR for initially diagnosed patients. We grouped the C_t values of positive specimens into 8 strata, and selected convenience samples in adequate numbers for each stratum to make the total sample size 50 (Main article, Figure 1). Selected individual SARS-CoV-2-positive specimens were pooled with different numbers of SARS-CoV-2-negative specimens to make 50 sets of pooled specimens in duplicate; the pool sizes of each set were 2, 4, 6, 8, 10, and 16. A total of 600 pooled specimens were prepared. To evaluate clinical specificity in SARS-CoV-2-negative pooled specimens, we randomly combined 16 specimens from 300 negative specimens and made 60 negative pooled specimens.

b. RNA extraction of pooled specimens

The following 3 automated RNA extraction systems were used: MagNa Pure 96 (Roche Diagnostics, https://www.roche.com), Real-prep (BioSewoom, www.biosewoom.com), and eMAG (bioMérieux, https://www.biomerieux.com). We followed the extraction protocol provided by each manufacturer with an input volume of 200 µL and elution volume of 50 µL

c. Real-time RT-PCR (rRT-PCR)

We performed rRT-PCR (PowerCheck 2019-nCoV) with all pooled specimens. The interpretation guideline by manufacturer for SARS-CoV-2 positivity was C_t cutoff $\leq$35 for single

specimen. However, we checked every amplified curve until the end of the 40 PCR cycles in this study. When either the *E* or *RdRp* gene was observed to have any amplified curve within the end of the amplification cycles, 40, we interpreted the result as positive for the pooled specimens. When no amplification curves were observed until the C_t value reached 40 for both genes, the result was interpreted as negative.

The PCR protocol that we used was based on the protocol by Corman et al. (*3*). The Korea Centers for Disease Control and Prevention (KCDC) also used laboratory-developed tests (LDT) based on the same protocol. All of these protocols use the same primer and probe sequences. The KCDC also evaluated pooled specimens with various pool sizes with their laboratory-developed tests, and similar results were obtained (data not shown). Although other protocols based on the same protocol might show similar sensitivities with pooled specimens, we strongly recommend that the optimal pool sizes should be evaluated for each protocol.

d. Statistical analysis

All statistical analyses were performed with MedCalc ver.19.2.1 (MedCalc Software Ltd, https://www.medcalc.org).

3. Protocol

Based on the results obtained, the COVID-19 task force of KSLM and the KCDC Center for Laboratory Control of Infectious Diseases has recommended the following protocol for molecular diagnostic testing of pooled specimens of upper respiratory specimens. This protocol is not recommended for use in other situations, such as the initial diagnosis of symptomatic patients. This protocol is aimed to maximize testing capacity with minimal loss of sensitivity in the following situations:

1) When rapid testing is critical for screening positive cases in large populations, e.g., screening the asymptomatic patients in the long-term care hospitals, or screening in the healthcare works without symptom.

2) When testing resources are limited. Because differences in specimen collection, nucleic acid extraction, and real-time RT-PCR reagent can affect sensitivities of pooled specimens, laboratories must validate the possible effect of them before using this protocol.

a. Specimen types

- Upper respiratory specimens including oropharyngeal and nasopharyngeal swab specimens are recommended to be used for this protocol.
- When lower respiratory specimens are required from patients in accordance with clinical or epidemiological findings, testing using individual specimens is recommended rather than pooled specimens.

b. Specimen container

- Virus transport media; for example, UTM, or
- Special media for virus molecular diagnostic testing; for example, guanidinium-based buffer, such as Copan eNAT, can be used.

  Special media for molecular diagnostic testing contain substances to inactivate the virus, which is beneficial for specimen handling. Pooling must be performed with the specimens of the same media type.

c. Specimen collection, transport, and general biological safety of the specimen

Upper respiratory tract specimens should be collected and handled in accordance with the KCDC (*4*,*5*) or KSLM (*1*) guidelines.

d. Specimen pooling

1) Determination of the number of specimens pooled and volumes for nucleic acid extraction

The total number of specimens to be pooled needs to be determined. The volume for nucleic acid extraction should be checked in accordance with the manufacturer’s instructions. The probability of obtaining positive results from the pooled samples is higher as more specimen volume is used for nucleic acid extraction.

A predetermined dilution factor should be selected after careful consideration of the characteristics of the targeted population and the expected disease prevalence. We recommend

the pooling of ≤6 specimens. If the 96% sensitivity is tolerable, the pooling of ≤10 specimens might be considered.

2) Protocol for specimen pooling

a) It is recommended for the personnel performing the specimen pooling to wear appropriate personal protective equipment (N95, KF94, or equivalent or superior respiratory protectors, full-body gown, gloves, etc.) and to process specimens, using a ≥Class II biosafety cabinet (BSC) in a biosafety level 2 facilities. Despite processing specimens in a molecular diagnostic testing tube with the inactivation substance, the same procedure should be followed. When specimens are processed outside of the BSC, personal protective equipment, including an N95 respiratory mask or other superior-quality respiratory protectors, must be worn. After processing, the safety cabinet should be sterilized with 70% isopropyl alcohol or equivalent.

b) Before pooling, the specimens should be thoroughly vortexed for ≥30 s in a tightly closed container, using a vortex mixer.

c) Each specimen should be placed in a new tube for pooled specimens. Equal volumes should be transferred using a sterile disposable pipette tip. The new tube for pooled specimens should be the same as the tube for each specimen. (See b. Specimen Container.) When opening the lid of the original specimen container, care should be taken to not contaminate the surroundings.

d) The total number of pooled specimens should be determined such that they do not exceed 60% of the container volume.

e) When specimens are pooled through aliquoting, sterile disposable pipette tip or droppers (disposable plastic transfer pipette) should be used.

f) When using a sterilized dropper, the tips need not be changed, as opposed to when using a pipette, and there is a lower chance of cross-contamination. Therefore, it can be useful for large-scale testing. Only sterilized droppers (e.g. sterilization with ethylene oxide gas) should be used. The recommended volume dispensed from a dropper is 500 μL. A 15-mL conical tube can be used as a transfer container, and the volume should be noted as described in d). After aliquoting all specimens, they should be vortexed for >10 s and the necessary volume should be harvested for extraction.

g) When pooling aliquots using a micropipette, equal volumes from each specimen should be transferred into a new container and mixed. For each specimen or at each step, a new pipette tip should be used. An accurate specimen volume can be collected with a micropipette. However, with an increased number of pooled specimens, the volume required for collection from each tube decreases, leading to an increased chance of error. Therefore, calculations should be performed with more than 10% overfill to prepare pooled specimens (Table 1). Numbers in Table 1 can be modified when preparing other volumes of pooled specimens.

e. Nucleic acid extraction

- Nucleic acid extraction from the pooled specimens should be performed in accordance with the protocol of the manufacturers of the nucleic acid extraction reagent/kit or the test reagents.
- When using a device with high extraction efficiency and when using more specimen volume for nucleic acid extraction, chances of viral detection are higher in pooled specimens. Therefore, the number of specimens used for nucleic acid extraction and the eluted amount and concentration of the extracted nucleic acid should be confirmed before using the device.
- An automated extraction device is advantageous for efficient extraction of numerous specimens.

f. Test implementation

- Tests will be performed in accordance with the manufacturer's guidelines, using RNA extracted from pooled specimens.
- This protocol is based on the results by PowerCheck 2019 nCoV Real-Time Detection Kit, emergency use authorization version (Kogene Biotech). Any modification of the protocol should be verified by each laboratory before implementation. The extent of its application can be modified in accordance with future scientific evidence.

g. Interpretation of the test results using pooled specimens

Based on COVID-19 laboratory guidelines, the test result from an individual patient should be considered positive only when all genes targeted in the diagnosis kit are detected (*1*,*6*). However, this protocol is aimed at screening positive specimens from multiple patients and not at making a confirmed diagnosis (Table 2).

Therefore, when using pooled specimens, the amplification curve for each gene should be visually assessed, and when an amplification is observed within the maximum cycle set by the manufacturer <u>for any one of the genes</u>, **the outcome should be considered presumptive positive.** Molecular testing should be performed for each individual specimen in the pool to determine positive or negative outcomes.

The results obtained for specimens, where there is an absence of amplification of all genes and absence of amplification of the internal quality control for pooled specimens should be considered invalid. Testing of individual specimens would be determined accordingly for subsequent testing.

h. Interpretation of the results of pooled samples tests

When an amplification curve within the maximum cycle set by the manufacturer is observed from any one of the genes from pooled specimens, each specimen should be individually tested.

Based on the results of testing individual specimens, positive, undetermined, or negative results should be decided, and appropriate action should be subsequently taken. Details regarding the tests for individual specimens and its interpretations should follow the KSLM-KCDC COVID-19 Laboratory Diagnostic Guidelines and the guidelines of the manufacturers of the reagents (*1*,*6*).

References

1. Hong KH, Lee SW, Kim TS, Huh HJ, Lee J, Kim SY, et al. Guidelines for laboratory diagnosis of coronavirus disease 2019 (COVID-19) in Korea. Ann Lab Med. 2020;40:351–60. PubMed https://doi.org/10.3343/alm.2020.40.5.351

2. Korea Centers for Disease Control and Prevention. Guidelines in response to coronavirus disease 2019 (for local government). Osong-eup, Cheongwon-gun, Republic of Korea: Centers for Disease Control and Prevention; 2020.

3. Corman VM, Landt O, Kaiser M, Molenkamp R, Meijer A, Chu DK, et al. Detection of 2019 novel coronavirus (2019-nCoV) by real-time RT-PCR. Euro Surveill. 2020;25:2000045. PubMed https://doi.org/10.2807/1560-7917.ES.2020.25.3.2000045

4. Korea Centers for Disease Control and Prevention. Guidelines for collecting clinical specimens for 2019 novel coronavirus. Osong-eup, Cheongwon-gun, Republic of Korea: Centers for Disease Control and Prevention; January 23, 2020.

5. Korea Centers for Disease Control and Prevention. Interim guidelines for collecting, handling, and testing clinical specimens from persons for coronavirus disease 2019 (COVID-19). Osong-eup, Cheongwon-gun, Republic ok Korea: Centers for Disease Control and Prevention; 2020.

6. Sung H, Roh KH, Hong KH, Seong MW, Ryoo N, Kim HS, et al. COVID-19 Molecular testing in Korea: practical essentials and answers from experts based on experiences of emergency use authorization assays. Ann Lab Med. 2020;40:439–47. PubMed https://doi.org/10.3343/alm.2020.40.6.439

Appendix Table 1. Examples of a pooled specimen volume based on a 200-µL standard specimen volume for nucleic acid extraction

Specimen volume needed for nucleic acid extraction, µL	Total no. specimens in pool	Specimen volume to collect for each original specimen, µL	Total specimen volume, µL
200	2	110	220
200	4	60	240
200	6	40	240
200	8	30	240
200	10	25	250

Appendix Table 2. Suggested interpretation of the results of pooled specimens

E gene C_t value	*RdRp* gene C_t value	Interpretation and measures
Below cutoff	Below cutoff	Positive results on pooled samples screening (SARS-CoV-2 genes detected in the pooled specimens)<br>Individual specimens should be tested
Amplification within the maximum cycles predetermined per the manufacturer's protocol for >1 genes but not satisfying the standard for positive results for pooled specimens		Undetermined results on pooled samples screening (Possibility of SARS-CoV-2 genes in the pooled specimens)<br>® Individual specimens should be tested
Amplification beyond the maximum cycles predetermined per the manufacturer's protocol is NOT observed for either gene		Negative results on pooled samples screening (Absence of SARS-CoV-2 genes in the pooled specimens)

Considerations for Specimen Pooling for SARS-CoV-2 Virus RT-PCR

Recommendations for Adaptation of Authors' Protocol

This article is based on the opinion of authors. We discussed the factors related to design, the experiment, and guideline development. The opinions expressed in the supplemental data by authors do not necessarily reflect the opinions of the Korea Centers for Disease Control and Prevention or the institutions with which the authors are affiliated.

1. Analytical Performance and Characteristics of RT-PCR Kits
    A. Analytical sensitivity (limit of detection) is a critical factor for the performance of pooling specimens. Poor sensitivity can cause false negatives, causing disastrous situations. Therefore, for the adaptation of the RT-PCR kit to the pooling strategy, the analytical sensitivity of the intended kit should be evaluated.
    B. Analytical specificity should also be evaluated for the possibility of false-positive results, as it will influence the efficiency, including cost-effectiveness.
    C. Most countries have their own systems for the Emergency Use Authorization (EUA) or regular approval of in vitro diagnostic (IVD) products. We highly recommend using IVD kits approved by the domestic EUA or Regular System.
    D. When using multiplex PCR kits, the criteria for interpretation of pooled specimens should be determined before using. The kits use variable target genes and show variable amplification efficiency, and their sensitivity among genes may differ.
    E. The threshold for fluorescence, the cutoff for C_t values, and the maximum amplification cycles should be considered together to determine the maximum pool size.
2. RNA Extraction Method
    A. The purity of RNA and efficiency of the RNA extraction method may influence the results. Therefore, the RNA extraction method should be validated in a laboratory with the selected RT-PCR kit.
    B. One of the main factors determining the final volume for pooling is the sample input volume used for RNA extraction. The most frequently recommended volume for RNA extraction by commercial kits is approximately 200 μL. Some systems can be used with larger volumes up to 1 mL. Users can validate various protocols with different sample volumes to determine the efficiency, cost-effectiveness, and performance of the test.

3. Type and Quality of Specimens

   A. When making the specimen pool, the volume from each sample should be the same. To obtain the same amount of the specimen, the specimen should be homogenous and liquid. Therefore, upper respiratory specimens in media are good for pooling, in contrast to sputum.

   B. Regarding the specimen buffer, to avoid the matrix effect, we recommend using the specimens with same specimen buffer for pooling.

   C. After pooling, to make the pooled sample homogenous, rigorous vortexing is required.

4. Pooling Method
   There might be three types of pooling methods. We recommend using media pooling for efficiency and ease of handling.

   A. Swab Pooling

      i. Method: The swabs from different individuals are acquired and mixed in the same sample tube.

      ii. Characteristics

         1. This method avoids the decrease in sensitivity. Multiple are in the same volume of transport media with that of individual testing.

         2. The amount of background human nucleic acid could be increased.

         3. The number of sample tubes used is reduced.

         4. The possibility of hazard to laboratory personnel by splashing or environmental contamination during specimen processing is highest among the three methods.

         5. When positive results are obtained in the pooling test, sample collection from each patient in the pool should be performed again. It might be necessary for the patient to return to healthcare facility. Besides, the viral load in the patient might change during disease course.

   B. Media Pooling

      i. Method: Swab acquisition is done separately in each sample tube. After submission to the laboratory, the same amount of media is taken from each tube and mixed for RNA extraction.

      ii. Characteristics

         1. After a positive result in the pooling test, confirmation of the positive result with each specimen can be performed easily.

2. The C_t value of pooled specimen is higher than single positive specimen. False negative results may occur if too many specimens are pooled.

3. Usually, there is no need to resample from individuals for confirmation.

4. Laboratory contamination during pooling is less likely.

C. RNA Pooling

i. Method: Sample acquisition is done separately in each sample tube. After submission to the laboratory, RNA extraction is performed separately. The extracted RNA is pooled and used for PCR.

ii. Characteristics

1. This method might avoid the interference of inhibitors in the samples.

2. Only the PCR tests are saved by pooling. All the sample tubes and RNA extraction steps are the same with individual testing.

3. The concerns about C_t value changes and false negative results exist as media pooling.

4. After positive results in pooling tests, PCR can be performed easily.

5. Usually, there is no need to resample for confirmation

5. Using the Cumulative Sensitivity for the Validation of the Test with Pooled Specimens

A. Maintaining sensitivity is important during pooling; therefore, we used cumulative sensitivity for the validation steps.

B. Based on the cumulative sensitivity and specificity of the kit used, the pool size should be chosen carefully. We recommended choosing pool sizes from six to ten.

6. Clinical Considerations for Efficiency

A. Prevalence of COVID-19 in the Population for Surveillance

i. The efficiency of pooling is mainly determined by the prevalence of COVID-19 and pooling size. Therefore, the laboratory should consider the expected prevalence before performing tests with pooled samples.

B. Number of Specimens for Pooling

i. Based on the performance of the RNA extraction kit and RT-PCR kit, the number of pooled specimens should be limited.

C. Convenience of Resampling

i. Swab pooling may be suitable in some instances. The laboratory should consider

the convenience of resampling, as swab pooling needs resampling..

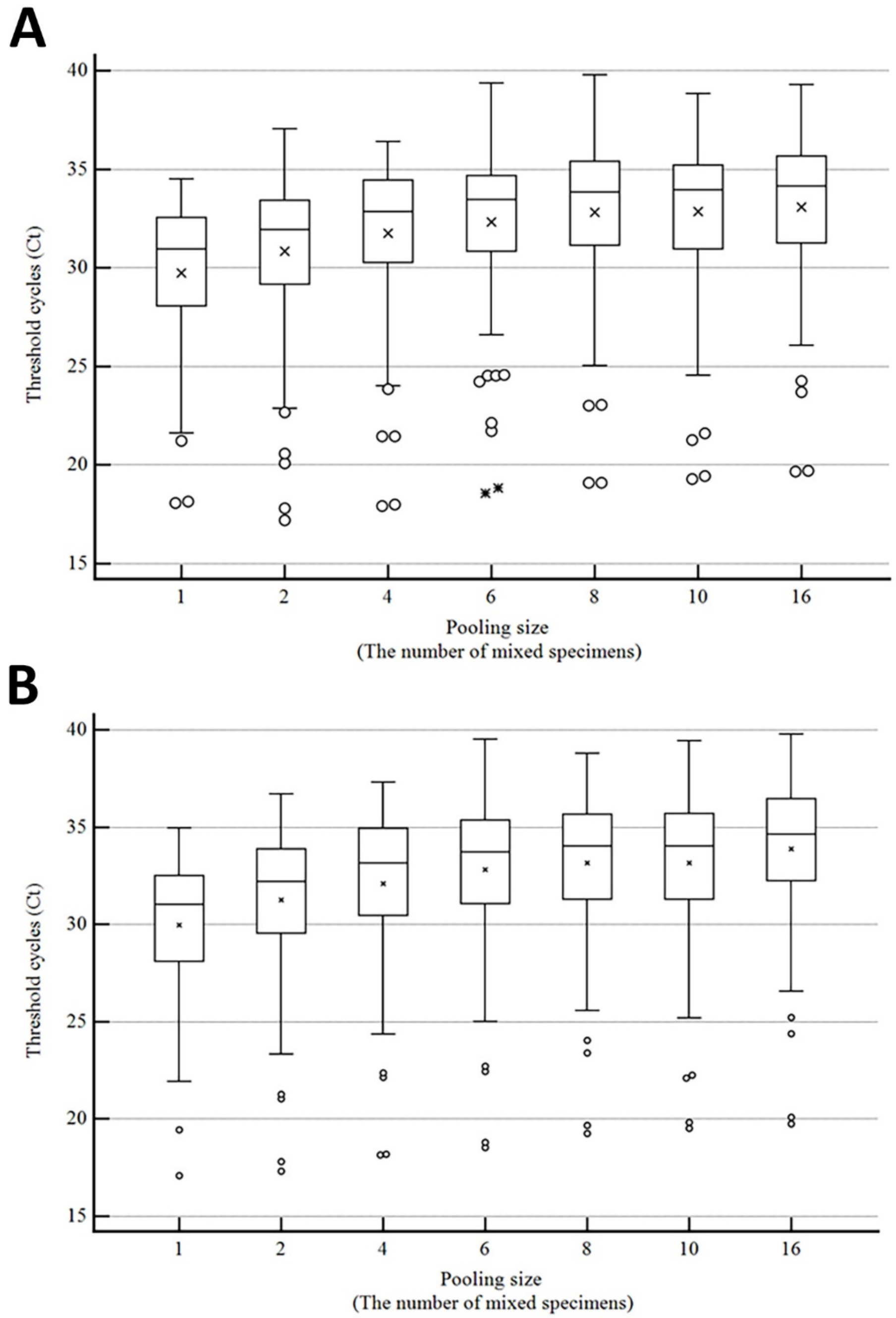

Appendix Figure. Distribution and mean threshold cycle (C_t) of the *E* and *RdRp* genes in each pool size. (A) *E* gene and (B) *RdRp* gene. X indicates mean C_t values. Open circles represent results outside of 95% CI of the cluster based on the median.
